# Upper tract urothelial carcinoma in Germany: epidemiological data and surgical treatment trends in a total population analysis from 2006 to 2019

**DOI:** 10.1007/s00345-022-04219-5

**Published:** 2022-11-29

**Authors:** Roman Herout, Martin Baunacke, Luka Flegar, Angelika Borkowetz, Alina Reicherz, Rainer Koch, Klaus Kraywinkel, Christian Thomas, Christer Groeben, Johannes Huber

**Affiliations:** 1 Department of Urology, University Hospital Carl Gustav Carus, TU Dresden, Dresden, Germany; 2 Department of Urology, Philipps-University Marburg, Marburg, Germany; 3 Department of Urology, Ruhr-University of Bochum, Marien Hospital Herne, Herne, Germany; 4 National Center for Cancer Registry Data, Robert Koch Institute, Berlin, Germany

**Keywords:** Radical nephroureterectomy, Endoscopic therapy, Ureteral tumor, Renal pelvis tumor, Health services research

## Abstract

**Purpose:**

To report contemporary epidemiological data and treatment trends for upper tract urothelial carcinoma (UTUC) in Germany over a 14-year period.

**Methods:**

We analyzed data from the nationwide German hospital billing database and the German cancer registry from 2006 to 2018/2019. The significance of changes over time was evaluated via regression analysis. Survival outcomes were calculated using the Kaplan–Meier method.

**Results:**

There was a non-significant increase in the age-standardized incidence rate from 2.5/100,000 in 2006 to 2.9/100.000 in 2018. 13% of patients presented with lymph node metastasis and 7.6% of patients presented with distant metastasis at primary diagnosis. The 5-year overall survival was estimated at 45% and the 10-year overall survival at 32%. Endoscopic biopsies of the renal pelvis and ureter as well as ureteroscopies with excision/destruction of UTUC all increased significantly over the study period. The number of radical nephroureterectomies (RNU) for UTUC steadily increased from 1643 cases in 2006 to 2238 cases in 2019 (*p* < 0.005) with a shift from open surgery towards minimally invasive surgery. Complex reconstructive procedures like ileal ureter replacement or autotransplantation are rarely performed for urothelial carcinoma of the ureter.

**Conclusion:**

Diagnostic and therapeutic procedures for UTUC have increased and minimally invasive nephroureterectomy is the predominant approach concerning radical surgery in 2019.

**Supplementary Information:**

The online version contains supplementary material available at 10.1007/s00345-022-04219-5.

## Introduction

About 5–10% of all urothelial carcinomas (UC) are located in the ureter or collecting system of the kidney [1]. Upper tract urothelial carcinoma (UTUC) is a rare disease with an estimated incidence of 1–2 per 100 000 person-years in Western countries [1]. However, in a recent publication from the Norwegian cancer registry a substantially higher age-standardized incidence rate (ASR) of 4.7 per 100.000 from 2014 to 2018 was reported [2].

For high-risk UTUC (multifocal disease, tumors > 2 cm, high-grade tumors, local invasion on CT, hydronephrosis, previous radical cystectomy for high-grade bladder cancer, variant histology) radical nephroureterectomy with bladder cuff excision is still considered as the gold standard [3]. Kidney-sparing approaches have historically been limited to patients with imperative indications (solitary kidney, bilateral UTUC, severely impaired kidney function), but technological advances in the endoscopic armamentarium have led to significant improvements in organ-sparing UTUC management [4–6]. Additionally, studies have shown that distal and segmental ureterectomy can achieve comparable oncologic outcomes to radical nephroureterectomy in carefully selected patients [7, 8]. Total ureterectomy with ileal ureteric replacement and kidney autotransplantation after ureterectomy remain further surgical treatment options at highly specialized centers and limited to well-selected patients [9–11]. The aim of this study was to report current epidemiological data as well as surgical treatment trends regarding UTUC in Germany.

## Materials and methods

### German hospital billing database (diagnosis-related groups)

A total population analysis of the nationwide billing data in Germany from 2006 to 2019 was performed.

Health insurance, either statutory or private, is mandatory for all citizens and permanent residents (PRs) in Germany. Citizens or PRs enroll in either so-called “sickness funds”, which is basically the statutory health insurance (SHI), or in private health insurance if certain criteria pertaining to income are met. The hospitals are reimbursed for their inpatient services by these nongovernmental health insurance plans (SHI) or private health insurance and reimbursement is regulated by the diagnosis-related groups (DRG). These DRGs are comprised of an ICD-10 (International Statistical Classification of Diseases and Related Health Problems) diagnosis code and an OPS (German adaption of the International Classification of Procedures in Medicine) code for the performed intervention. Local data are transferred to the Institute of Hospital Remuneration and subsequently to the German Federal Statistical Office (Destatis). With the exemption of patients treated exclusively in psychiatric, forensic, and military hospitals, the nationwide Destatis database contains every reimbursed inpatient case in Germany and can thus be regarded as complete for the given purpose. Our group has described the methodology used for this work in detail [12].

Specific ICD-10 codes for upper tract urothelial carcinoma were combined with OPS codes for diagnostic and therapeutic procedures to assess UTUC-specific surgeries. Detailed information on the methodology is provided in the supplements.

### German National Centre for Cancer Registry data at the Robert Koch Institute

Epidemiological data on cancer diagnosis from each German state is centrally merged at the German National Centre for Cancer Registry Data at the Robert Koch Institute. Nationwide incidence for 3-digit ICD-10 diagnoses (here: C65 and C66), starting from the year 1999, is regularly estimated using mixed Poisson regression models to account for regional and/or temporal underreporting of cases. The Revised European Standard Population (2013 ESP) was used to determine the ASR. To include non-invasive UTUC (Ta and Tis), incidence for these tumors (ICD-10: D09.1) was separately estimated using the ratio of observed invasive/non-invasive cases in each age group and calendar year. Survival was calculated using the Kaplan–Meier method, using data from five selected German cancer registries that fulfilled predefined quality indicators. Cancer-specific survival (CSS) was estimated by calculating relative survival by dividing the overall survival after UTUC diagnosis by the survival as observed in the German population of the same age and sex.

### Data protection and ethics statement

We followed the "REporting of studies Conducted using Observational Routinely collected health Data" (RECORD) statement and performed all actions in accordance with the Declaration of Helsinki in its latest version [13]. Analyzed data were completely anonymized and derived from established databases with rigorous data protection measures. Therefore, an additional ethics statement was not required.

### Statistics

Linear regression for trend analysis and Wald tests were performed with SAS V9.4 (SAS Institute, Cary, NC).

## Results

### Epidemiology

The incidence of UTUC in Germany increased steadily with estimated case numbers of 2089 in 2006 and 2693 cases in 2018 (*p* < 0.03 for trend analysis). Likewise, we observed a non-significant increase in the ASR from 2.5/100,000 in 2006 to 2.9/100.000 in 2018 (*p* = 0.2). Figure S1 depicts the incidence stratified by age, comparing the years 2006 and 2018. The age group between 80 and 84 years showed the highest incidence rates. In figure S2 the age and sex-specific incidence rates per 100.000 persons are shown for the entire study period.

**Fig. 1:**
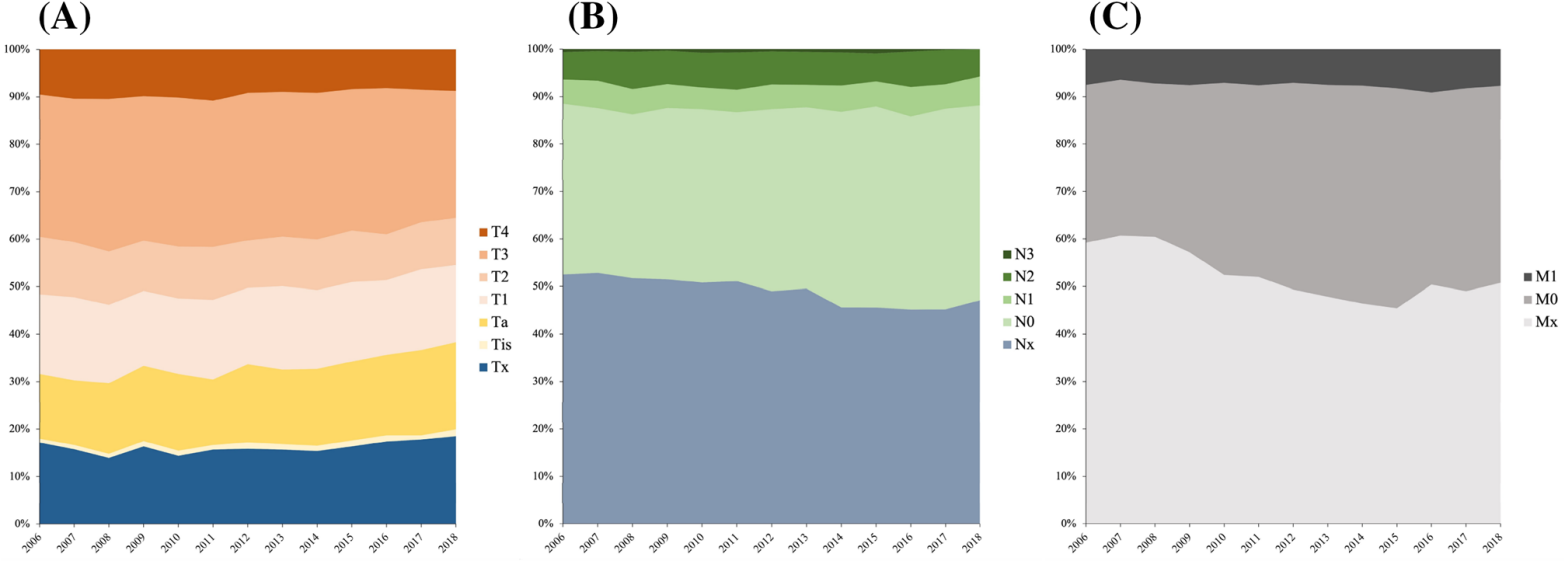
Development of tumor stages (**A**), lymph node stages (**B**) and distant metastasis (**C**) at first diagnosis of upper tract urothelial carcinoma in Germany

Overall, 13% of patients presented with lymph node metastasis and 7.6% of patients presented with distant metastasis at primary diagnosis. Figure 1 shows the development of the incidence of tumor stages, lymph node status and metastatic disease. The 5-year overall survival (OS) was estimated to be 45% and the 10-year OS was 32%. The corresponding 5-year cancer-specific survival (CSS) was estimated to be 54.7% and the 10-year CSS 48.5%. We observed a small, but steady decline in OS over time from 46.9% (patients diagnosed in 2003–2005, 95%-CI 44.2–49.6%) to 43.7% (2012–2014, 95%-CI 41.2–46.1%). Likewise, a decline in CSS rates was noticed, from 57% (2003–2005) to 53.5% (2012–2014).

### Diagnostic procedures

All described trends refer to the study period from 2006 to 2019 unless another time frame is specified. As shown in Figure S3, we observed an increase in transurethral biopsies of the renal pelvis (for all indications) from 606 to 1731 cases (*p* < 0.001; + 186%). Similarly, we observed an increase in transurethral biopsies of the ureter (for all indications) from 1682 to 4128 (*p* < 0.001; + 154%). The number of biopsies of the renal pelvis specifically performed for UC of the renal pelvis increased from 191 to 568 cases (*p* < 0.001; increase of 197%). The mean age of patients undergoing a biopsy of the renal pelvis for UC increased from 69.9 to 71.7 years (*p* < 0.001). Biopsies of the ureter specifically for UC of the ureter increased from 312 to 873 cases (*p* < 0.001; increase of 180%) and the mean age of the patients increased from 71.2 to 72.6 years (*p* = 0.001). 65.5% of all patients undergoing transurethral biopsy for UC were male and 34.5% were female and the gender distribution remained stable over the study period.

### Therapeutic procedures

The number of ureteroscopies with excision or destruction of urothelial carcinoma in the renal pelvis increased from 222 to 358 cases (*p* = 0.003 increase of 61%). The mean age of patients undergoing this procedure increased from 69.3 to 73.1 years (*p* < 0.001). Likewise, the number of ureteroscopies with excision or destruction of UC located in the ureter increased from 222 in 2009 cases to 461 in 2019 (*p* < 0.003; increase of 108%) with the mean age of patients increasing from 72.1 to 73.9 years (*p* < 0.02).

In the study period, an average of 16 patients were treated percutaneously for upper tract urothelial carcinoma per year with a mean age of patients of 71.8 years.

The case numbers of partial ureterectomies for UTUC increased from 154 to 254 (*p* = 0.002; increase of 65%). The total number of partial ureterectomies for all indications (including benign disease) was 1417 in 2019 in Germany, hence 18% of all partial ureterectomies in 2019 were performed for UTUC.

A total of 3851 nephroureterectomies were performed in Germany in 2019. Of these, 2238 were coded in combination with UC (58%). The number of nephroureterectomies for UTUC steadily increased over the study period from 1643 to 2238 cases (*p* = 0.005; increase of 36%). The mean age of patients undergoing nephroureterectomy increased from 70 to 71.9 years (*p* < 0.001). In 2019, 1398 nephroureterectomies were performed for the UC of the renal pelvis (62%) and 874 for the UC of the ureter (38%). In 702 of the 2238 nephroureterectomy cases, a bladder cuff was coded as well (31%).

**Fig. 2:**
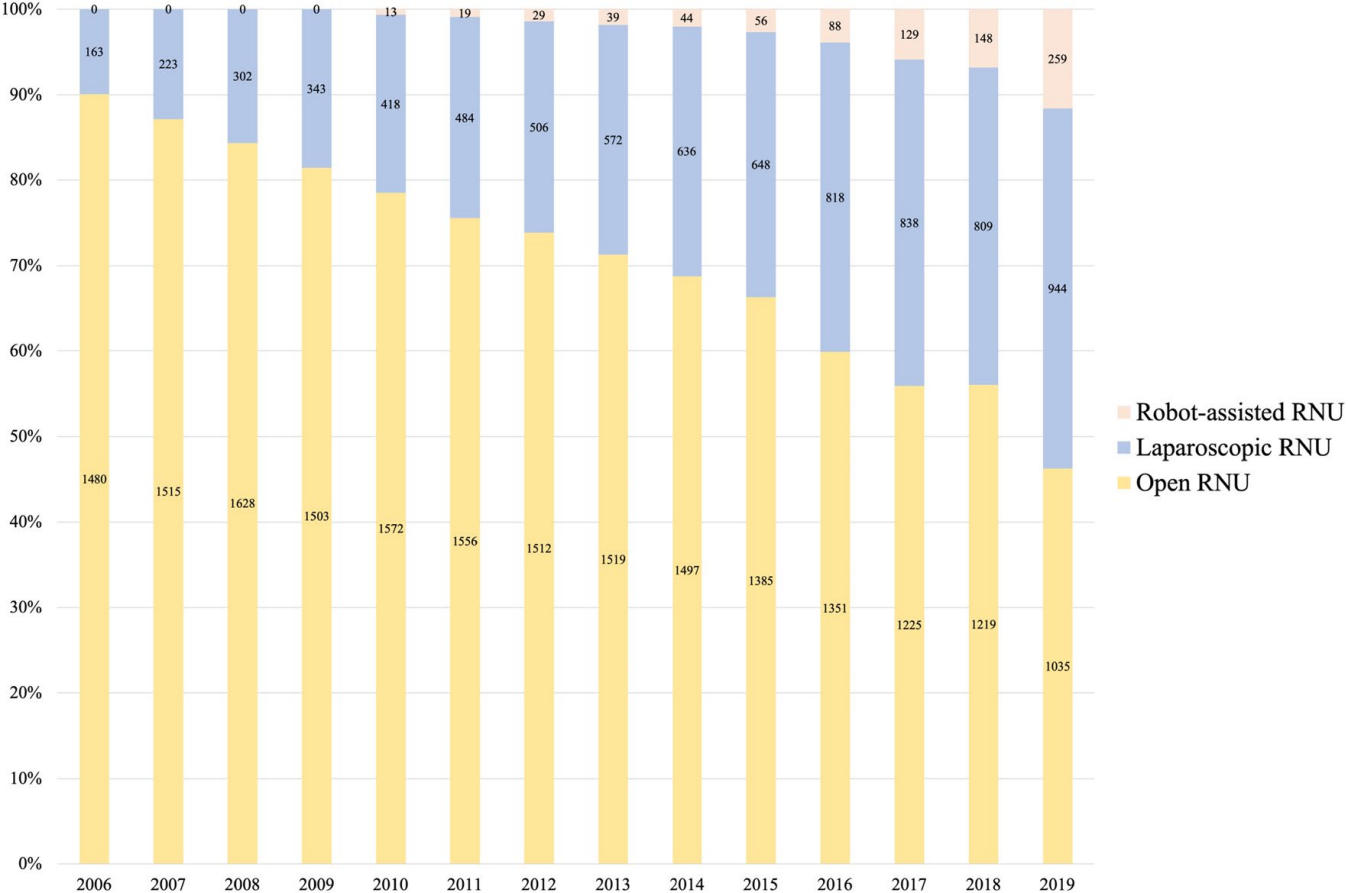
Radical nephroureterectomy (RNU) in Germany: distribution of open, laparoscopic and robot-assisted surgery between 2006 and 2019

The number of laparoscopic nephroureterectomies for all indications steadily increased from 316 cases to 1387 (*p* < 0.001; increase of 339%). Likewise, the number of laparoscopic nephroureterectomies for UTUC increased from 163 cases to 944 (*p* < 0.001; increase of 479%). The number of clinics that performed laparoscopic nephroureterectomies increased from 69 to 211 (*p* < 0.001; increase of 206%). Likewise, the number of robotic radical nephroureterectomies for UTUC increased from 13 cases in 2010 to 259 cases in 2019 (*p* = 0.001; increase of 1892%), as shown in Fig. 2. The number of clinics that performed this robotic procedure for UTUC increased from 4 in 2010 to 69 in 2019 (*p* < 0.001; increase of 1625%).

The ratio of radical nephroureterectomies to ureteroscopic therapies shifted from 5.1:1 in 2006 to 2.5:1 in 2019, representing a distinct increase in ureteroscopic therapies.

The number of cases where a cytotoxic agent was instilled in the urinary bladder after nephroureterectomy increased from 3 cases in 2006 to 133 cases in 2019, accounting for a total of 5.9% of all patients after nephroureterectomy in 2019.

Case numbers for ileal ureter replacement for UTUC remained steady and low throughout the study period with 3–13 cases per year (*p* = 0.3). A total of 7 cases of renal autotransplantation after resection of the ureter for UTUC were recorded in Germany in the study period.

## Discussion

In this study we present the first contemporary data on the epidemiology as well as current trends regarding surgical interventions for upper tract urothelial carcinoma in Germany.

According to data obtained from the national cancer registry, we observed an estimated increase of 28.9% in UTUC cases over the study period. The age-standardized rate (ASR) adjusted to the European standard population, did not significantly increase with an ASR of 2.5 in 2006 and 2.9 in 2018 (*p* = 0.2; increase of 1.38% per year). In a recent publication by Almås et al., data from the Norwegian cancer registry showed considerably higher ASRs of 3.9 for the time period 1999–2018 and 4.7 for 2014–2018 [2]. In contrast, Ruvolo et al. reported data extracted from the SEER-18 registry (data from 2004 to 2016 comprising 34.6% of the US population), where they found a significant decline in age-standardized incidence rates from 1.3 per 100 000 person-years to 1.1 per 100 000 person-years in the US [14]. However, their findings were not in accordance with earlier observations made by Raman et al., when they utilized data from the SEER-9 registry (data from 1973 to 2005, comprising 10% of the US population) and found an increase in the ASR from 1.9 in 1973 to 2.1 in 2005 [15].

In our study, a stage shift towards organ-confined disease was observed, with an increasing proportion of non-invasive UTUC cases (pTa, pTis, pT1) from 31 to 36% and declining case numbers of muscle-invasive and advanced disease (pT2-pT4) from 52 to 46% from 2006 to 2018. This shift towards lower stages was accompanied by a change in surgical approach with more patients being treated ureteroscopically compared to radical nephroureterectomy. Almås et al. observed a decline of invasive tumors from 50 to 42% that was not statistically significant, whereas Ruvolo et al. reported a decrease in localized and an increase in metastatic UTUC in the US population [2, 14]. The number of patients with nodal-positive disease in the presented work remained stable at 13%, although the true number might be higher since almost 50% of patients were classified as Nx.

The 5-year OS was estimated to be 45% and the 10-year OS 32%. In the most recent work regarding UTUC survival by Almås et al., the authors reported a 5-year OS of 48% and a 10-year OS of 33%, which are comparable survival rates to our findings [2]. In general, 5-year OS in documented UTUC cohorts ranged between 32 and 50% [15–17]. We noticed a decline in OS and CSS over time by approximately 4%, when the years 2003–2005 and 2012–2014 were compared. This deterioration in OS and CSS is most likely not attributable to an artifact, such as improved data quality, but might represent a true decline in survival. However, the reason for this trend is unknown and a possible link between increasing numbers of ureteroscopic therapies and decreased survival rates cannot be verified with the given data.

We observed an increase of 186% in ureteroscopic biopsies of the ureter and renal pelvis for all indications over the study period. With an ageing population and the highest incidence in the age group of patients 80–84 years, we furthermore detected a statistically significant trend towards older patients undergoing diagnostic and therapeutic procedures for UTUC.

In contrast to the report of Almås, we found rising case numbers of radical surgery, with 1643 radical nephroureterectomies performed in 2006 and 2238 in 2019. Laparoscopic and robot-assisted laparoscopic radical nephroureterectomy cases significantly increased over time. In 2006 only 10% of nephroureterectomies were performed laparoscopically and no robot-assisted procedures were documented. However, in 2019 minimally invasive nephroureterectomy accounted for 54% of all nephroureterectomies and in conformity with these findings, the number of clinics that offered these minimally invasive approaches remarkably increased as well, with 69 clinics offering laparoscopic nephroureterectomy in 2006 and 211 clinics in 2019. Likewise, robot-assisted radical nephroureterectomy was offered in 4 clinics in 2010 and 69 clinics in 2019. The initial hesitancy to adopt minimally invasive surgery for UTUC could have been triggered by concerns about the oncologic safety with regard to the increased intra-abdominal pressure due to the capnoperitoneum and subsequent tumor seeding. This has triggered an ongoing debate in the urological community. However, due to advances in technique (not entering the urinary tract and *en bloc* removal of kidney, ureter and bladder cuff as well as the use of endobags) and careful patient selection, published data did not confirm worse oncologic outcomes of patients with organ-confined disease treated with the laparoscopic vs. open surgery [18]. Current recommendations in the European guidelines consider invasive and large T3/T4 tumors with lymph node or distant metastasis as a contraindication for minimally invasive surgery, as data have shown that the minimally invasive approach might be associated with worse oncological outcomes in patients with advanced UTUC [19, 20]. So, given that 54% of patients underwent minimally invasive radical nephroureterectomies in 2019 and about 40% of patients initially present with advanced or high-risk disease we assume that most patients suitable for a minimally invasive approach nowadays undergo laparoscopic or robot-assisted laparoscopic radical nephroureterectomy in Germany.

The code for bladder cuff excision (BCE) was only used in 31% of radical nephroureterectomies. However, we cannot report on the exact percentage of patients who underwent bladder cuff resection due to the nature of the billing data. We assume that the low percentage of BCE during RNU might be a blur in the billing system, since coding the BCE is not linked to pecuniary rewards in the DRG-based reimbursement system and may therefore be underutilized.

Instillation of Mitomycin-C (MMC) was shown to reduce the rate of intravesical urothelial carcinoma recurrence in two prospective, randomized trials and was subsequently recommended as an adjuvant treatment in the 2017 update of the European guidelines on UTUC [21–23]. We observed a steady and significant incline of cases where a cytotoxic agent was instilled into the urinary bladder after radical nephroureterectomy. However, documented instillations were still very low in 2019 (6% of all RNU). Like with bladder cuff excision, a specific OPS code for the instillation of cytotoxic substances into the urinary bladder does exist but is not linked to higher reimbursement. We therefore cannot make a valid proposition with regard to postoperative bladder recurrence prophylaxis.

Finally, only very few patients underwent major reconstructive surgery, i.e. ureterectomy combined with ileal ureter replacement and nephrectomy with autotransplantation for UTUC. These challenging procedures do not play a major role in the surgical treatment of ureteral UC in Germany.

We present comprehensive data on the incidence, survival and surgical treatment modalities concerning UTUC in Germany. Given the rarity of the disease, we were able to provide reliable data on surgical treatment options by using billing data that comprise almost all surgically treated UTUC cases in Germany. However, a few shortcomings must be addressed. Data obtained from the DRG billing database is highly accurate, however, clinical information on tumor and patient characteristics are limited. Also, rigorous data protection does not allow to identification of single patients or institutions from the DRG database. Generally, data collection for UTUC via the DRG database was complex as many procedural (OPS) codes are non-UTUC specific and partially redundant. Due to the nature of the data, we do not have detailed information on lymphadenectomies and on the surgical options pertaining to the management of the distal ureter and bladder cuff during radical nephroureterectomy. In addition, we do not have data on chemotherapy and immunotherapy for this patient cohort, thus the report solely focusses on the operative management of the disease.

## Conclusion

In the present study, we observed a significant increase in surgical therapies for UTUC while the ASR did not significantly increase over the study period. With regards to endoscopy, case numbers of biopsies of the ureter and kidney for UTUC as well as therapeutic ureteroscopies significantly increased over the study period. A dramatic shift towards minimally invasive nephroureterectomy was observed with 54% of patients undergoing either laparoscopic or robot-assisted laparoscopic surgery in 2019.

## Supplementary Information

Below is the link to the electronic supplementary material.

Supplementary file1 (DOCX 1998 KB)

## Data Availability

All data used in this work are stored centrally at the specific institutes (German Federal Statistical Office – Destatis; German National Centre for Cancer Registry Data at the Robert Koch Institute). The datasets used and analyzed for this work are available from the corresponding author on request.

## References

[1] Siegel RL, Miller KD, Fuchs HE, Jemal A (2021). Cancer statistics, 2021. CA Cancer J Clin.

[2] Almås B, Halvorsen OJ, Johannesen TB, Beisland C (2021). Higher than expected and significantly increasing incidence of upper tract urothelial carcinoma. A population based study. World J Urol.

[3] Rouprêt M, Babjuk M, Burger M (2021). European association of urology guidelines on upper urinary tract urothelial carcinoma: 2020 update. Eur Urol.

[4] Daneshmand S, Quek ML, Huffman JL (2003). Endoscopic management of upper urinary tract transitional cell carcinoma: long-term experience. Cancer.

[5] Rouprêt M, Hupertan V, Traxer O (2006). Comparison of open nephroureterectomy and ureteroscopic and percutaneous management of upper urinary tract transitional cell carcinoma. Urology.

[6] Yakoubi R, Colin P, Seisen T (2014). Radical nephroureterectomy versus endoscopic procedures for the treatment of localised upper tract urothelial carcinoma: a meta-analysis and a systematic review of current evidence from comparative studies. Eur J Surg Oncol.

[7] Lughezzani G, Jeldres C, Isbarn H (2009). Nephroureterectomy and segmental ureterectomy in the treatment of invasive upper tract urothelial carcinoma: A population-based study of 2299 patients. Eur J Cancer.

[8] Kato T, Nakayama R, Haba T (2018). Oncological and renal outcomes of segmental ureterectomy vs. radical nephroureterectomy for upper tract urothelial carcinoma. Oncol Lett.

[9] Banerji JS, George AJP (2014). Total ureterectomy and ileal ureteric replacement for TCC ureter in a solitary kidney. Can Urol Assoc J.

[10] Ou Y-C, Hu C-Y, Cheng H-L, Yang W-H (2018). Long-term outcomes of total ureterectomy with ileal-ureteral substitution treatment for ureteral cancer: a single-center experience. BMC Urol.

[11] Janssen MWW, Linxweiler J, Philipps I (2018). Kidney autotransplantation after nephrectomy and work bench surgery as an ultimate approach to nephron-sparing surgery. World J Surg Oncol.

[12] Groeben C, Koch R, Baunacke M (2016). Robots drive the German radical prostatectomy market: a total population analysis from 2006 to 2013. Prostate Cancer Prostatic Dis.

[13] Benchimol EI, Smeeth L, Guttmann A (2015). The reporting of studies conducted using observational routinely-collected health Data (RECORD) Statement. PLOS Med.

[14] Collà Ruvolo C, Nocera L, Stolzenbach LF (2021). Incidence and survival rates of contemporary patients with invasive upper tract urothelial carcinoma. Eur Urol Oncol.

[15] Raman JD, Messer J, Sielatycki JA, Hollenbeak CS (2011). Incidence and survival of patients with carcinoma of the ureter and renal pelvis in the USA, 1973–2005. BJU Int.

[16] Eylert MF, Hounsome L, Verne J (2013). Prognosis is deteriorating for upper tract urothelial cancer: data for England 1985–2010. BJU Int.

[17] Woodford R, Ranasinghe W, Aw H (2016). Trends in incidence and survival for upper tract urothelial cancer (UTUC) in the state of Victoria - Australia. BJU Int.

[18] Piszczek R, Nowak Ł, Krajewski W (2021). Oncological outcomes of laparoscopic versus open nephroureterectomy for the treatment of upper tract urothelial carcinoma: an updated meta-analysis. World J Surg Oncol.

[19] Peyronnet B, Seisen T, Dominguez-Escrig J-L (2019). Oncological outcomes of laparoscopic nephroureterectomy versus open radical nephroureterectomy for upper tract urothelial carcinoma: an european association of urology guidelines systematic review. Eur Urol Focus.

[20] Simone G, Papalia R, Guaglianone S (2009). Laparoscopic versus open nephroureterectomy: perioperative and oncologic outcomes from a randomised prospective study. Eur Urol.

[21] O’Brien T, Ray E, Singh R (2011). Prevention of bladder tumours after nephroureterectomy for primary upper urinary tract urothelial carcinoma: a prospective, multicentre, randomised clinical trial of a single postoperative intravesical dose of mitomycin C (the ODMIT-C Trial). Eur Urol.

[22] Ito A, Shintaku I, Satoh M (2013). Prospective randomized phase II trial of a single early intravesical instillation of pirarubicin (THP) in the prevention of bladder recurrence after nephroureterectomy for upper urinary tract urothelial carcinoma: the THP Monotherapy Study Group Trial. J Clin Oncol.

[23] Roupret M, Babjuk M, Compérat E (2018). European association of urology guidelines on upper urinary tract urothelial carcinoma: 2017 update. Eur Urol.

