# Supplementary material for: Upper tract urothelial carcinoma in Germany: epidemiological data and surgical treatment trends in a total population analysis from 2006 to 2019
Source: World J Urol. 2022 Nov 29;41(1):127–33. doi: 10.1007/s00345-022-04219-5 (PMC9849285; doi:10.1007/s00345-022-04219-5)
Supplement: Supplementary file 1 — Supplementary file1 (DOCX 1998 KB) [file 345_2022_4219_MOESM1_ESM.docx]

**Supplements**

**Title: “Upper Tract Urothelial Carcinoma in Germany: Epidemiological Data and Surgical Treatment Trends in a Total Population Analysis from 2006 to 2019”**

**Authors:** Roman Herout^1^, Martin Baunacke^1^, Luka Flegar^2^, Angelika Borkowetz^1^, Alina Reicherz^3^, Rainer Koch^2^, Klaus Kraywinkel^4^, Christian Thomas^1^, Christer Groeben^2*^, Johannes Huber^2*^

^1^ Department of Urology, University Hospital Carl Gustav Carus, TU Dresden, Dresden, Germany

^2^ Department of Urology, Philipps-University Marburg, Marburg, Germany

^3^ Department of Urology, Ruhr-University of Bochum, Marien Hospital Herne, Herne, Germany

^4^ National Center for Cancer Registry Data, Robert Koch Institute, Berlin, Germany

Corresponding authors Email: [roman.herout@uniklinikum-dresden.de](mailto:roman.herout@uniklinikum-dresden.de)

**Supplementary methods**

ICD codes C65 (carcinoma of the renal pelvis), C66 (carcinoma of the ureter) and D091 (carcinoma in situ of other and unspecified urinary organs) were used to specifically address interventions for UTUC. To assess endoscopic biopsies of the renal pelvis, OPS code 14600 (transurethral biopsy of the renal pelvis) was first used separately and subsequently in combination with ICD code C65. Likewise, the code 14601 (transurethral biopsy of the ureter) was first analyzed alone and then in combination with C66. To assess ureteroscopic procedures we used OPS codes: 55522 (ureteroscopic excision of (diseased) tissue of the kidney), 55526 (ureteroscopic destruction of (diseased) tissue of the kidney), 55633 (ureteroscopic excision of (diseased) tissue of the ureter) and 55634 (ureteroscopic destruction of (diseased) tissue of the ureter). There are 3 different OPS codes for nephroureterectomy: 55545 – radical nephrectomy with ureterectomy, 55546 – radical nephrectomy with transurethral resection of the ureter (“pluck” technique) and 5554b – nephrectomy, with ureterectomy. Laparoscopic nephroureterectomy (OPS codes: 555453, 555463, 5554b3) and robot-assisted nephroureterectomy cases (code OPS5987: application of a complex operative robot) were obtained separately. The ratio of radical nephroureterectomies to endoscopic therapies for UTUC was calculated by dividing the number of radical nephroureterectomies [(C65 OR C66 OR D091) AND (55545 OR 55546 OR 5554b)] by the number of endoscopic therapies for UTUC [(C65 OR C66 OR D091) AND (55522 OR 55526 OR 55633 OR 55634)].

The OPS code 85414 (instillation of and locoregional therapy with cytotoxic and immunomodulatory substances into the urinary bladder) was used to determine cases that received a postoperative instillation of a cytotoxic agent as adjuvant treatment to lower the risk of bladder recurrence. To assess the number of open-surgery ileal ureter replacements after total ureterectomy as well as kidney autotransplantation for UTUC we combined the ICD code C66 with the specific code for ileal ureter replacement (5568g0) and autotransplantation of the kidney (55553).

**Supplementary figures**

(A) (B)


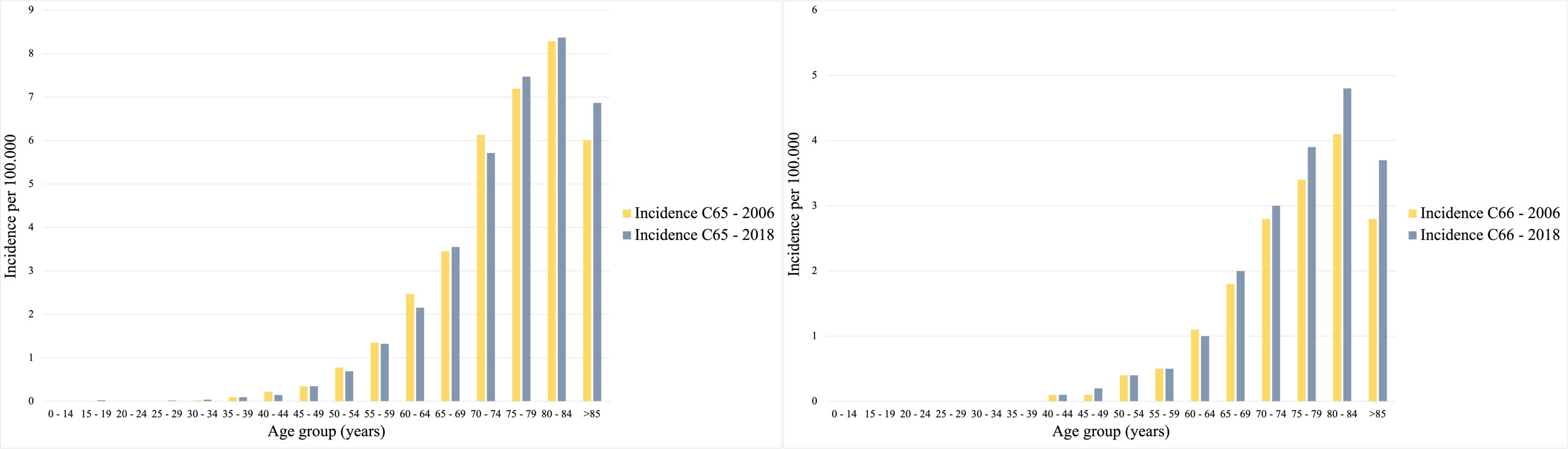


**Figure S1**. Incidence rates of invasive urothelial carcinoma of the renal pelvis – C65 (A) and the ureter – C66 (B) stratified by age. Yellow bars indicate the incidence in the year 2006 and gray bars in the year 2018.

***
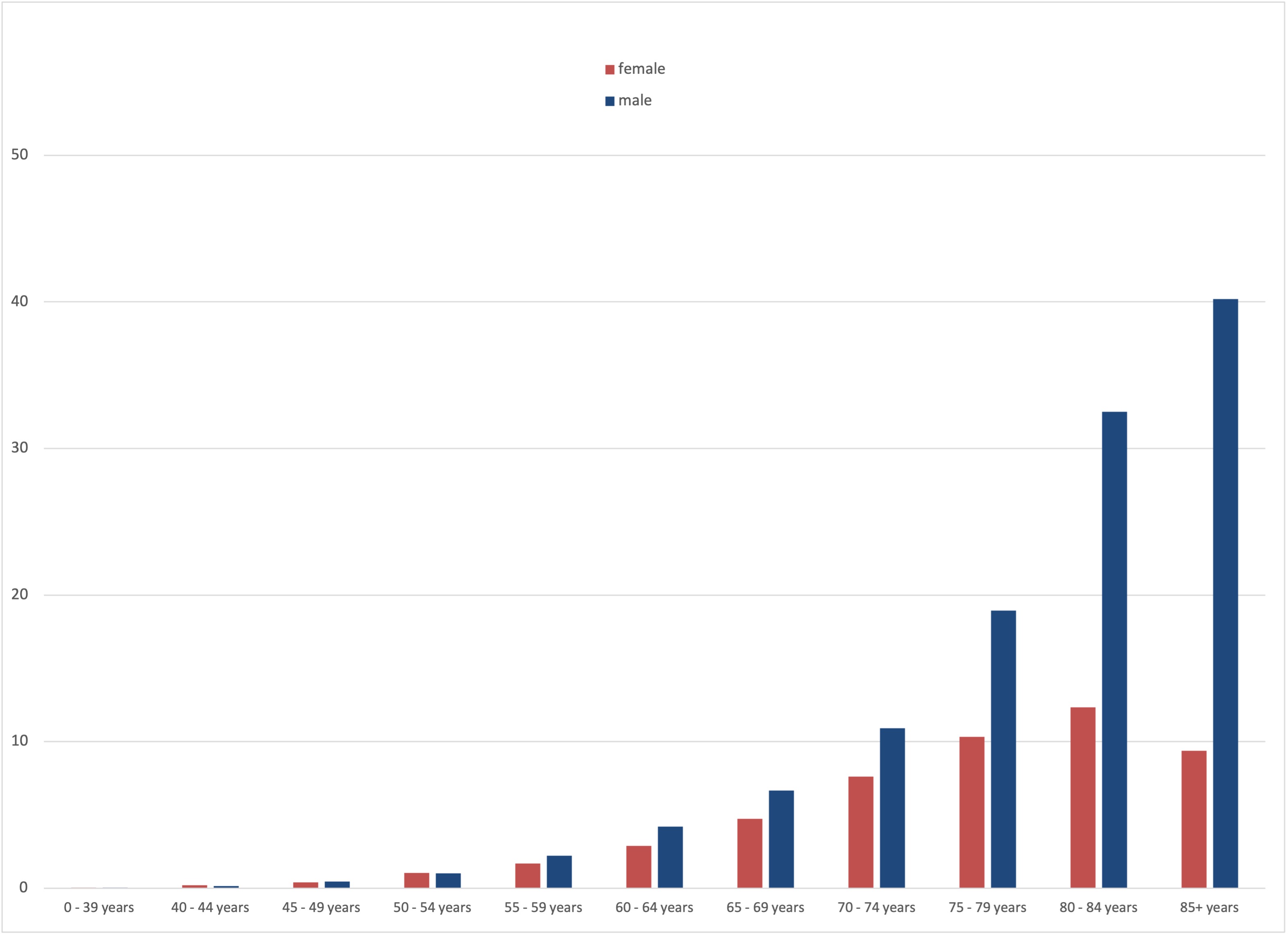
***

**Figure S2.** Age and sex specific incidence rates of UTUC (per 100.000 persons) in Germany from 2006 – 2018.


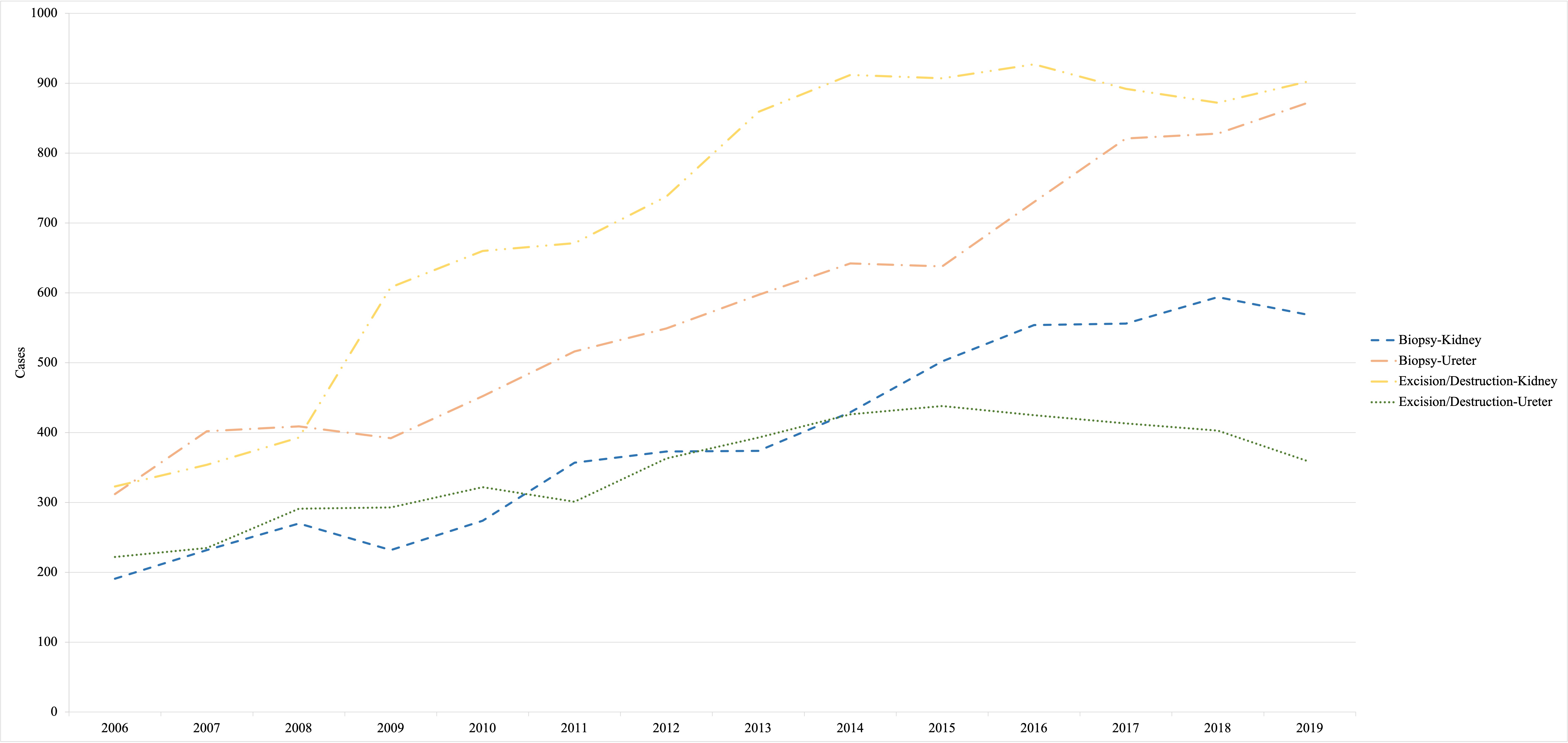


**Figure S3.** Case numbers of ureteroscopic procedures for UTUC in Germany.
